# 2314: Orexin/hypocretin receptor 2 (HCRTR2) in alcohol dependence diagnosis and severity: An exploratory investigation in the role of HCRTR2 rs2653349 polymorphism

**DOI:** 10.1017/cts.2017.39

**Published:** 2018-05-10

**Authors:** Tim D Klepp, Primavera Spagnolo, Pei-Hong Shen, Nancy Diazgranados, Colin Hodgkinson, Vijay Ramchandani, David Goldman

## Abstract

OBJECTIVES/SPECIFIC AIMS: The preliminary analysis sought to retrospectively characterize the role of hypocretin receptor 2 (HCRTR2) in the development and prognosis of AD along with associated behavioral measures including smoking, self-reported drinking history, and neuroticism. Given the results in this study along with the paucity of information regarding the functional significance of rs2653349, we intend to comprehensively characterize HCRTR2 using haplotype analyses. We will then identify relationships between our haplotype analysis and IV alcohol self-administration using the Computer-Assisted Infusion System, and phenotypes identified in a sleep study. Furthermore, we aim at identifying functional loci in the hypocretin/orexin system by investigating differential allele expression in the orexin receptors in hippocampus tissue obtained from postmortem human brains. METHODS/STUDY POPULATION: This study examined 1569 European American and African American individuals between 18 and 65 years old, 922 of whom with a current diagnosis of AD. Participants were genotyped for HCRTR2 rs2653349 and ancestry was determined via a genome-wide panel of ancestry informative markers. AD was diagnosed using the Structured Clinical Interviews for DSM-IV (SCID-IV) for psychiatric disorders and recent alcohol use was assessed by 90-day Timeline Follow-back (TLFB) interviews. Smoking was assessed using the Fagerström Test for Nicotine Dependence and neuroticism was measured using the NEO Personality Inventory. RESULTS/ANTICIPATED RESULTS: In European Americans, a significant difference was found in current AD diagnosis between AX carriers and GG carriers (*z*=−2.390, *p*=0.017). This relationship remained significant in a logistic regression model controlled for age and gender (*R* ^2^=0.269, *p*=0.015). TLFB drinking measures were compared based on the median values to correct for the ceiling effect resulting from the assessment covering the past 90 days. Total drinks (*U*=8.280, *p*=0.004), number of drinking days (*U*=6.983, *p*=0.008), and average drinks per days (*U*=7.221, *p*=0.007) were all noted to significantly differ between the two allele groups among Caucasians. The associations between rs2653349 and total drinks (*R* ^2^=0.115, *p*=0.023) and heavy drinking days (*R* ^2^=0.190, *p*=0.015) remained significant in linear regressions controlled for age and gender. Furthermore, Caucasian AX carriers had a higher median number of drinking days relative to GG homozygotes among current AD positive subjects (*U*=6.937, *p*=0.012) and a lower median number of drinking days among current AD negative subjects (*U*=4.430, *p*=0.035). Among Caucasian AD negative subjects, there was a significantly greater frequency of smokers (χ^2^=3.550, *p*=0.046). In African American participants, there were no significant differences in AD diagnosis and in measures of AD severity by genotype. African American males diagnosed with current AD had higher rates of smoking in the AX group (χ^2^=4.969, *p*=0.017). No significant associations were found between rs2653349 and neuroticism in any of the cohorts analyzed in this sample. DISCUSSION/SIGNIFICANCE OF IMPACT: The results suggest that, among Caucasians, AX carriers have an increased risk to develop AD independently of their age and gender. In addition, among individuals with a diagnosis of AD, AX carriers reported a greater number of drinking days, as measured by the TLFB, suggesting that this polymorphism also exerts an effect on the severity of the disease. This effect on increased alcohol consumption was absent in Caucasian AX carriers without current AD diagnosis. In future analysis, we will explore how different genetic profiles in HCRTR2, and also HCRTR1, may alter the orexin signaling pathway and how such alterations may predispose patients to develop AD and exacerbate AD once it develops.

